# App Engagement as a Predictor of Weight Loss in Blended-Care Interventions: Retrospective Observational Study Using Large-Scale Real-World Data

**DOI:** 10.2196/45469

**Published:** 2024-06-07

**Authors:** Marco Lehmann, Lucy Jones, Felix Schirmann

**Affiliations:** 1 Oviva AG Potsdam Germany; 2 Oviva UK Limited London United Kingdom

**Keywords:** obesity, weight loss, blended-care, digital health, real-world data, app engagement, mHealth, mobile health, technology engagement, weight management, mobile phone

## Abstract

**Background:**

Early weight loss is an established predictor for treatment outcomes in weight management interventions for people with obesity. However, there is a paucity of additional, reliable, and clinically actionable early predictors in weight management interventions. Novel blended-care weight management interventions combine coach and app support and afford new means of structured, continuous data collection, informing research on treatment adherence and outcome prediction.

**Objective:**

Against this backdrop, this study analyzes app engagement as a predictor for weight loss in large-scale, real-world, blended-care interventions. We hypothesize that patients who engage more frequently in app usage in blended-care treatment (eg, higher logging activity) lose more weight than patients who engage comparably less frequently at 3 and 6 months of intervention.

**Methods:**

Real-world data from 19,211 patients in obesity treatment were analyzed retrospectively. Patients were treated with 3 different blended-care weight management interventions, offered in Switzerland, the United Kingdom, and Germany by a digital behavior change provider. The principal component analysis identified an overarching metric for app engagement based on app usage. A median split informed a distinction in higher and lower engagers among the patients. Both groups were matched through optimal propensity score matching for relevant characteristics (eg, gender, age, and start weight). A linear regression model, combining patient characteristics and app-derived data, was applied to identify predictors for weight loss outcomes.

**Results:**

For the entire sample (N=19,211), mean weight loss was –3.24% (SD 4.58%) at 3 months and –5.22% (SD 6.29%) at 6 months. Across countries, higher app engagement yielded more weight loss than lower engagement after 3 but not after 6 months of intervention (*P*_3 months_<.001 and *P*_6 months_=.59). Early app engagement within the first 3 months predicted percentage weight loss in Switzerland and Germany, but not in the United Kingdom (*P*_Switzerland_<.001, *P*_United Kingdom_=.12, and *P*_Germany_=.005). Higher age was associated with stronger weight loss in the 3-month period (*P*_Switzerland_=.001, *P*_United Kingdom_=.002, and *P*_Germany_<.001) and, for Germany, also in the 6-month period (*P*_Switzerland_=.09, *P*_United Kingdom_=.46, and *P*_Germany_=.03). In Switzerland, higher numbers of patients’ messages to coaches were associated with higher weight loss (*P*_3 months_<.001 and *P*_6 months_<.001). Messages from coaches were not significantly associated with weight loss (all *P*>.05).

**Conclusions:**

Early app engagement is a predictor of weight loss, with higher engagement yielding more weight loss than lower engagement in this analysis. This new predictor lends itself to automated monitoring and as a digital indicator for needed or adapted clinical action. Further research needs to establish the reliability of early app engagement as a predictor for treatment adherence and outcomes. In general, the obtained results testify to the potential of app-derived data to inform clinical monitoring practices and intervention design.

## Introduction

The rise of digital technology enables new treatments for people with overweight or obesity. Digital care elements increasingly augment weight management interventions, resulting in a variety of different digitally supported modes of care [1]. Some modes use digital technology as a communication tool between patients and clinicians; other variants provide stand-alone digital care. A third variant uses digital elements to enhance clinicians’ care, blending digital and human care. This so-called blended care has been shown to deliver beneficial health effects [2] and holds the promise of scalability due to its partially digital character and remote mode of delivery [3].

Apps are frequently used for blended-care interventions, mainly due to the ubiquity of smartphones. Accordingly, a host of app-based blended-care interventions for weight management has emerged, spurring research on their efficacy. Several studies indicate their effectiveness in supporting people with overweight or obesity in weight management [1,4,5]. Furthermore, these interventions have additional benefits. Remote delivery simplifies access to care, which is especially relevant for underserved populations [6]. Also, they can increase the uptake of referred patients (to start the intervention) [7] and adherence to the intervention [8].

Moreover, blended care enables the collection of additional, otherwise inaccessible, data originating from patients’ interactions with health apps. The generated data are continuous and dense, potentially representing the entire length of an intervention with a plethora of data points. In addition, the data can be passively collected, that is, it is a by-product of patients’ interaction with a given app that reduces the burden of data collection on patients (eg, through questionnaires) and offers an extra source of information (eg, on top of patient reports). Thus, digitally derived data could support remedying the dearth of research data relating to nutrition [9].

Connected to this is the growing availability of real-world evidence from app-based blended-care interventions, affording large-scale analyses of weight management interventions [10]. As the reproducibility of real-world evidence has recently been documented, there is growing trust in its reliability [11]. Accordingly, using real-world evidence side-by-side with evidence from randomized controlled trials for the evaluation of interventions is becoming feasible [12]. Research on weight management interventions is profiting from real-world evidence due to the great magnitude of the data sets as well as the density, continuity, and high ecological validity of the data. Moreover, real-world evidence enables in-depth analyses (post hoc, but also live) on how patients respond to an intervention. For example, a large-scale, real-world data analysis using a digital app without face-to-face support recently revealed significant effects of usage frequency on weight loss in the short- and midterm for up to 4 months of treatment [10].

The mentioned usage frequency or, more broadly, patients’ engagement with an app is a key indicator of their treatment adherence [8]. Adherence to digital interventions has been succinctly defined as “[a] composite measure encompassing time online, activity completion, and active engagements with the intervention” [13]. However, various definitions of adherence (and its relation to engagement) coexist [14] and, in light of this complexity, standardization of the concept has been called for [15].

Nonetheless, app engagement is considered to be a valid indicator of adherence as well as a predictor of intervention outcomes [8], and app usage data assumes a pivotal role in terms of the measurability of engagement [16]. As high dropout rates (often measured as complete cessation of engagement) impact app-based interventions [17], researching engagement as a predictor of prospective adherence and anticipated treatment success is critical to inform intervention monitoring and to, potentially, trigger countermeasures preventing dropout. Regarding weight management interventions, several engagement metrics have been identified as predictors of overall weight loss. For example, self-weighing, persistent food logging, and activity were found to be significant predictors of weight loss during a 6-month intervention [18]. Using a point-based incentive system in a single-arm study, overall app engagement was shown to be associated with weight loss in a 3-month period [19]. Coaching, self-monitoring, and self-management were identified to be positively correlated with weight loss at 3 and 6 months [20]. Notably, the predictors seem to be time-sensitive, that is, their predictive value varies as per the time period in the treatment trajectory. Accordingly, time-dependent engagement, for example, in the first month, can be applied to inform the modeling of usage trajectories [21].

Against this backdrop, this retrospective study analyzes time-dependent app engagement as a predictor for weight loss in large-scale, real-world, blended-care settings. We hypothesized that patients who frequently used the app (higher engagers, ie, showing comparably higher engagement with the educational content, logging meals, and completing tasks) lose more weight than patients who use the app less frequently (lower engagers, ie, showing comparably lower engagement) at 3 and 6 months of a weight management intervention. Our analysis of real-world data aims to aggregate known indicators of app engagement into a standardized metric to predict weight loss. Furthermore, propensity score matching will allow us to isolate the causal effect of this metric of app engagement on weight loss in the 3- and 6-month periods. Finally, we provide separate analyses of structured weight management programs reimbursed in 3 different European health systems, enabling a first cross-country comparison of blended-care weight management interventions.

## Methods

### Study Design

This observational study used longitudinal real-world patient data from obesity treatment in weight management programs and obesity therapy in Switzerland, the United Kingdom, and Germany. Patients living with obesity used a mobile app (Oviva app [Oviva AG]) along with their treatment by dietitians and health coaches, known as the blended-care approach [22]. The study aimed to demonstrate the significant impact of using the mobile app (ie, engagement) on weight loss after 3 and 6 months of treatment. The data used for the analysis were automatically collected through the use of the app in the course of the treatment. Demographic data were collected in face-to-face, introductory, internet-based consultations and were recorded in a patient management system. There was no experimental manipulation involved, so the design yielded real-world data. The timeframe of enrollment was from January 3, 2019, to August 25, 2022. This report complies with the STROBE (Strengthening the Reporting of Observational Studies in Epidemiology) statement for transparent reporting of observational studies (STROBE checklist in Multimedia Appendix 1).

### Weight Management and Obesity Treatment Programs

The blended-care approach in Switzerland, the United Kingdom, and Germany similarly offers personalized support by dietitians and health coaches and combines this with digital care using the Oviva app. The app logs patient-reported weight, food, and activity data and keeps this information as a personal diary for the patient. It further provides learning content to empower patients to engage in a healthier lifestyle. The app also offers messaging between the patient and the dietitian or health coach. In all countries, treatment starts with an introductory consultation with a dietitian or health coach. However, there are also notable differences between countries. In Switzerland, patients are enrolled in a weight management program lasting 36 weeks. This consists of alternating coaching sessions and app-based messaging. Coaching sessions are structured interactions consisting of anamnesis, knowledge exchange, and providing patients with different options for weight reduction (eg, focus on meal size, snacks, and unhealthy beverages). Compliance with the intervention is monitored by the coaches and success is evaluated in the subsequent consultations. Patients from the United Kingdom are enrolled in the Tier 3 weight management program Way to Wellness (patients in obesity class 2 and above [BMI>35 kg/m^2^]). The program consists of 12 months of personalized support by the dietitians and health coaches. Patients can choose between app-based chat or telephone consultations weekly or biweekly for the first 16 weeks, with 30 minutes of monthly follow-up over 12 months. The topics and curriculum covered align with the international obesity guidelines, incorporating goal setting, action planning, healthy eating and weight loss strategies, problem-solving, lapse and relapse management, and maintaining behavior change. German patients are enrolled in an app-supported individual dietetic therapy for obesity lasting 3 to 6 months. The program consists of an introductory consultation of 30 minutes to identify the patient’s needs. Then, 4 further appointments of 15 to 30 minutes are scheduled within 6 months. Coaching sessions are personalized to cater to the individual patients’ therapeutic needs (eg, fostering nutritional literacy). Contact with the coach is further possible through app-based messaging. If patients are willing to continue after the first 5 appointments, a represcription to extend the treatment is possible.

### Participants

All patients were enrolled in the blended-care weight management program of their respective countries with the treatment aim of weight loss. Upon enrollment to the treatment program, patients provided written informed consent that their data stored in the Oviva app could be used for scientific purposes. Generally, patient access to the program was possible through a general practitioner referral. However, patients could sign up online with Oviva, declare their interest in the therapy, and then provide the referral later. In the United Kingdom, patients with BMI≥35 kg/m^2^ were eligible for participation in the Tier 3 weight management program. Besides referral, access to the program was also possible through self-referral using a prescription prepayment certificate maintained by the National Health Service. In Switzerland, patients with BMI≥25 kg/m^2^ were eligible, thus allowing patients with overweight and obesity in the program. In Germany, patients may enroll in the obesity therapy program after referral from their general practitioner. Eligibility here is not bound to a BMI threshold but depends on the respective health insurers’ policy for reimbursement of the treatment. After completing the program, patients may receive another referral to continue with the program.

### Measures

#### Primary Outcome: Percentage Weight Loss

**Figure 1:**
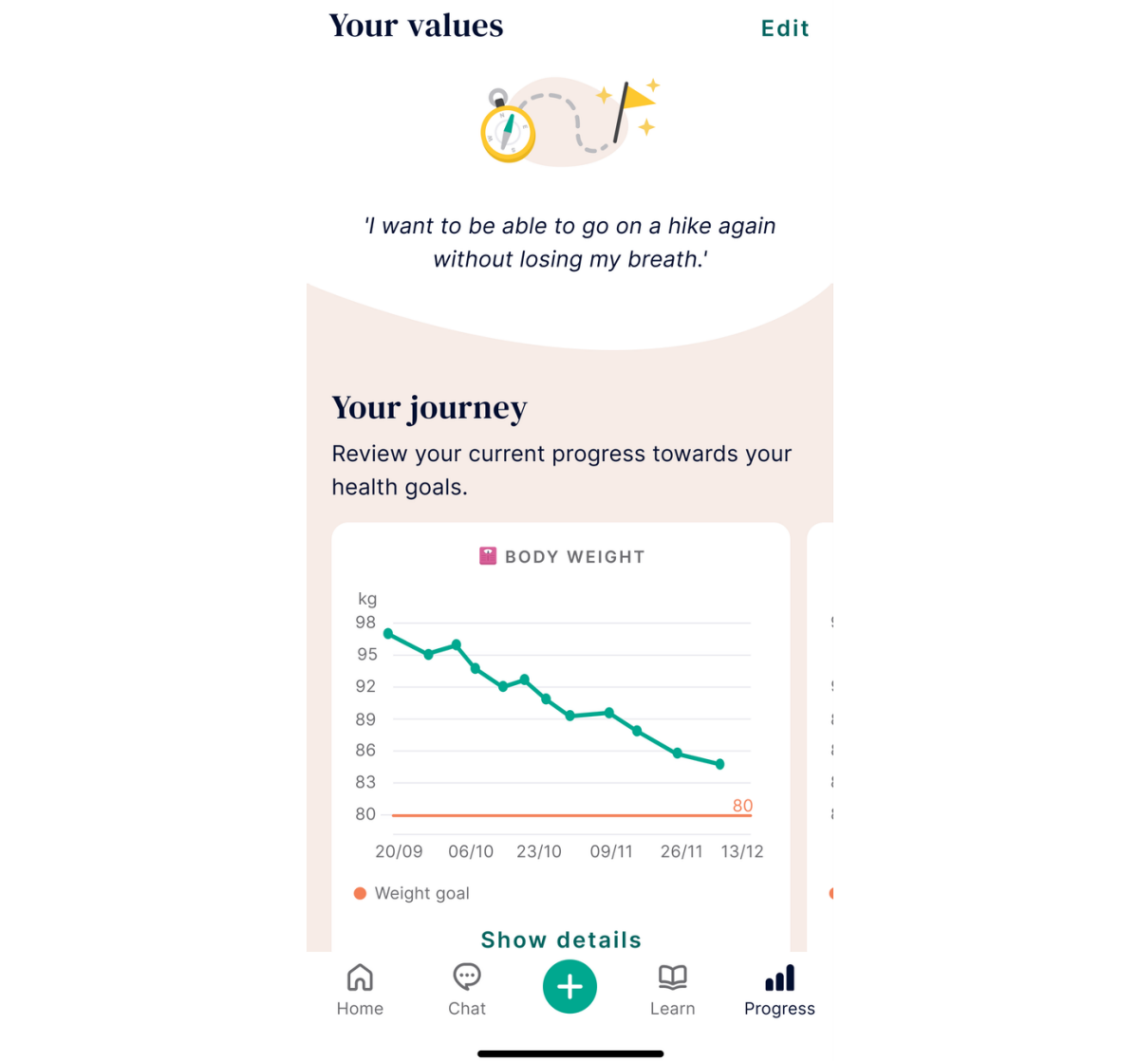
Oviva app showing development of body weight logs.

Patients used their own devices to measure their body weight and then entered the measurements in the Oviva app as weight logs (patient-reported outcomes; Figure 1). There was no limit regarding the frequency of weight logging in a given time frame. To derive percentage weight loss as the primary outcome, body weight was collected at baseline and at 3 and 6 months after baseline.

#### App Engagement

App engagement was measured using 5 indicators: meal logs, activity logs, weight logs, the number of completed goal-oriented tasks, and the number of access pages of learn content (Table 1). As part of the Oviva therapy, patients are asked to log meals and activities; complete tasks; and use learning modules that provide important dietary, behavior change, and health information. Logging takes minimal patient effort, focusing on a 1-click principle, which is always visible in the user interface (Figure 1). The app then offers several categories for logging, including meals and activities. There are different specifiers for meals and activities, such as meal ingredients or the type of activity. However, to derive a general measure of app engagement, we only counted the number of entries and not the content of entries. Task completion was handled through the diary function of the app. Patients could set and commit themselves to fulfill tasks. Task completion was recorded in the diary. Finally, patients could access several courses of learning content, each comprising several pages of the educational material. We used the count of accessed pages in the analysis. Counts in the period from baseline to 3 months of participation were captured as 3-month app engagement, and counts in the period between 3 months and 6 months of participation were captured as 6-month app engagement. All indicators were count variables.

**Table 1:** Indicators of app engagement used in multiple regression.

Intervention element and indicator of app engagement			Description		Unit
**Self-monitoring**					
	Meal logs	Number of meals tracked through text or photo within the Oviva app		Count	
	Activity logs	Number of logged activities, symptoms, or measurements (eg, weight or blood glucose) tracked within the Oviva app		Count	
	Weight logs	Number of logged weights within the Oviva app		Count	
**Self-management**					
	Completed tasks	Number of completed tasks assigned by the coach or the patient, for example, track your meal today and make 5000 steps		Count	
**Education**					
	Learn content	Accessed pages of learn content		Count	

#### Other Covariates

Additional patient characteristics included in the analysis were gender, age, and the presence of an obesity diagnosis (*International Classification of Diseases, Tenth Revision* [*ICD-10*] obesity [E66]). These characteristics were gathered by the dietitians or health coaches as part of the program enrollment. Furthermore, messages sent to coaches and messages received from coaches were considered in the analysis. These covariates are automatically recorded in the patient management system.

### Statistical Analysis

#### Sample Description

Patient data were obtained from the Oviva database. We used all available data within the described time period from the 3 programs in the United Kingdom, Switzerland, and Germany. All patient data were anonymized for the analysis. We split the available data according to the 3 treatment pathways in Switzerland, Germany, and the United Kingdom. For each, we have shown patient characteristics and weight loss after 3 and 6 months of treatment. We further reported linear regression models with percentage weight loss after 3 and 6 months as outcomes. App engagement, message exchange, and further patient characteristics served as predictors. App engagement was used as a binary predictor distinguishing between lower and higher engagement.

#### Characteristics of Lower and Higher App Engagement

The 5 indicators of app engagement (ie, meal count, accessed learn pages, weight logs, activity count, and task completion) were log_10_ transformed to enhance the impact of differences in the lower end of the scale and to remove the impact of extreme counts. For example, the distinction between 10 and 50 accessed pages of learn content should have more impact on the analysis than the difference between 510 and 550 accessed pages. All indicators entered a principal component analysis (PCA), which yielded 1 engagement metric as the first principal component (PC). A median split was then applied to this metric to yield 2 levels of app engagement, identifying lower and higher engagers. This split enabled optimal propensity score matching for gender, age, obesity diagnosis, start weight, and message exchange [23,24].

#### Weight Loss Models

**Figure 2:**
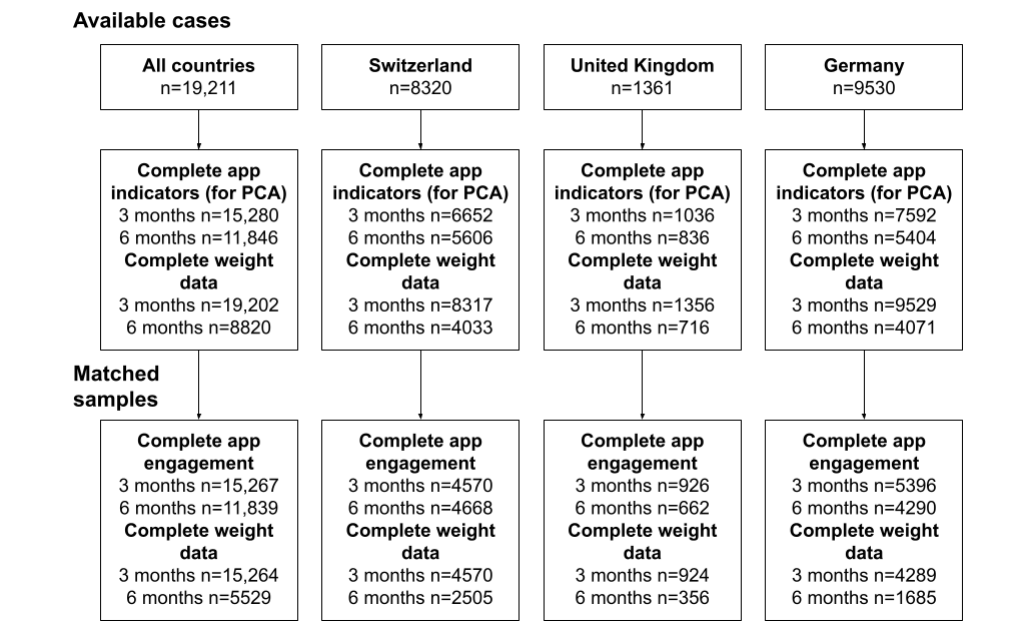
Flow diagram of available cases in the analysis. PCA: principal component analysis.

Multiple regression of weight loss after 3 and 6 months regarding lower and higher engagement and matched covariates will be reported. As main predictors, app engagement after 3 months and after 6 months were entered into the models. As covariates, we entered basic patient characteristics: gender, age, *ICD-10* E66 diagnosis (yes or no), and start weight. Furthermore, because the blended-care approaches in each of the 3 countries comprise digital patient-coach interaction, we further added the number of messages sent from or to the coach, respectively, as covariates. To make the 2 levels of higher and lower app engagement comparable, we used propensity score matching to achieve balanced data regarding the used covariates. So, in the regression models, there will be no confounding between app engagement and covariates. Matching was done separately for the 3- and 6-month periods and separately for the pooled data of all the countries and each of the 3 countries. This yielded 8 prespecified regression models to be reported. There was no covariate selection or removal using statistical criteria. Regression coefficients were tested using a 2-tailed *t* test at a level of significance α=.05. Statistical analysis used the R statistical software (R Core Team) [25]. Demographic information and descriptive statistics were aggregated using the *CreateTableOne* function from the *tableone* package [26], PCA used the *princomp* function from the *stats* package [25], propensity score matching used the *matchit* function (method parameter set to full matching or optimal matching) from the *MatchIt* package [23], and regression analyses used the *lm* function from the *stats* package [25]. All statistical methods used data from completed cases (Figure 2). We used no data imputation methods.

### Ethical Considerations

This study is completely based on anonymized observational data using no experimental manipulation or randomization. The Oviva app used by the patients is a registered Conformité Européenne–marked medical device according to the European Union Medical Device Regulation 2017/745 and is certified according to ISO 27001. As such, data collection was performed within the postmarket surveillance activities [27] and the app was used within its intended purpose by all patients. This study is not a clinical investigation according to Medical Device Regulation (MDR) 2017/745 [27] and there were no invasive or other burdensome methods applied. To use the app, patients gave informed consent that enables data analysis. Participants received no compensation. This study obtained no external funding.

## Results

### Sample Description

Data points from 19,211 patients (age range: 18-96 years) were available and were split according to the blended-care weight management interventions in 3 countries: individual dietetic therapy (Germany), app-supported dietary advice (Switzerland), and Tier 3 weight management (United Kingdom). A fraction of patients had no Oviva app available during their participation; these 2314 (12%) patients were excluded from further analyses. Table 2 shows the summary statistics of patient characteristics, weight loss, and indicators of app engagement. As can be seen, in all 3 countries, the majority (14,763/19,211, 76.9%) of patients were female and the mean age was 49.87 (SD 13.38) years. In Germany and Switzerland, the majority of patients had an *ICD-10* E66 diagnosis of obesity (Germany: 7281/9530, 76.4%; Switzerland: 5820/8320, 70%), whereas only a minority of patients in the United Kingdom had this diagnosis (354/1361, 26%). However, in the United Kingdom, patients were required to have >class 2 obesity (BMI≥35 kg/m^2^) to be eligible for the program, and this is also reflected in the much higher average start weight of these patients compared with the patients in Switzerland and Germany. For the entire sample (N=19,211), mean weight loss was –3.24% (SD 4.58%) at 3 months and –5.22% (SD 6.29%) at 6 months. Across countries, percentage weight loss for 3 and 6 months followed an expected pattern of –2.93% (–2.99 kg) to –4.44% (–5.6 kg) after 3 months of intervention and –4.68% (–4.78 kg) to –5.82% (–7.35 kg) after 6 months. Counts for messages and engagement indicators are not transformed in this table to allow for the appraisal of the average magnitude of these indicators. As can be seen, average meal counts are higher than counts of the 3 other indicators in both time periods, indicating the high importance of meal logging in obesity therapy.

**Table 2:** Patient characteristics.

Factor levels		All countries (N=19,211)	Switzerland (n=8320)	United Kingdom (n=1361)	Germany (n=9530)
**Gender, n (%)**					
	Male	4433 (23.1)	2691 (32.3)	288 (21.2)	1454 (15.3)
	Female	14,763 (76.9)	5629 (67.7)	1073 (78.8)	8061 (84.7)
Age (years), mean (SD)		49.87 (13.38)	50.19 (14.38)	48.63 (12.68)	49.77 (12.53)
**E66^a^ diagnosis, n (%)**					
	E66	13,455 (70.0)	5820 (70.0)	354 (26.0)	7281 (76.4)
	No diagnosis	1893 (9.9)	173 (2.1)	996 (73.2)	724 (7.6)
	Other diagnosis	3863 (20.1)	2327 (28.0)	11 (0.8)	1525 (16.0)
Start weight (kg), mean (SD)		100.53 (23.95)	94.48 (20.17)	126.23 (28.14)	102.13 (23.64)
**Percent weight loss, mean (SD)**					
	3 months	–3.24 (4.58)	–3.40 (4.93)	–4.44 (5.93)	–2.93 (3.98)
	6 months	–5.22 (6.29)	–5.65 (6.22)	–5.82 (7.37)	–4.68 (6.11)
**Messages from coach, mean (SD)**					
	3 months)	16.94 (22.65)	22.36 (29.00)	7.13 (16.58)	13.60 (14.50)
	6 months	8.35 (14.58)	11.40 (18.77)	1.44 (4.21)	6.67 (10.03)
**Messages to coach, mean (SD)**					
	3 months	2.34 (15.94)	2.44 (9.62)	11.35 (29.44)	0.97 (17.15)
	6 months	1.13 (15.10)	1.29 (6.46)	3.13 (8.19)	0.71 (20.32)
**Meal count, mean (SD)**					
	3 months	130.23 (117.51)	113.47 (90.53)	116.28 (80.28)	144.05 (135.54)
	6 months	130.17 (164.09)	123.03 (124.20)	110.02 (109.01)	137.54 (191.16)
**Activity count, mean (SD)**					
	3 months	48.35 (117.72)	34.86 (77.35)	33.66 (68.37)	57.01 (137.06)
	6 months	63.68 (166.85)	46.84 (109.12)	51.04 (121.31)	73.68 (192.72)
**Number of weight logs, mean (SD)**					
	3 months	13.57 (27.24)	10.60 (23.34)	9.64 (9.70)	16.72 (31.42)
	6 months	10.51 (23.18)	9.17 (23.54)	7.81 (12.78)	12.12 (23.93)
**Number of completed tasks, mean (SD)**					
	3 months	71.98 (96.53)	47.64 (57.23)	58.10 (69.70)	89.44 (114.27)
	6 months	76.17 (129.23)	54.66 (84.61)	65.00 (103.50)	90.87 (151.06)
**Pages of learn content, mean (SD)**					
	3 months	24.05 (31.13)	9.12 (15.59)	30.10 (35.89)	30.86 (33.59)
	6 months	18.04 (27.62)	6.61 (12.60)	19.41 (25.41)	24.26 (31.77)

^a^E66: obesity.

### Characteristics of Lower and Higher App Engagement

The 5 indicators of app engagement—meal count, activity count, weight logs, accessed pages of learn content, and the number of completed tasks—were aggregated with PCA to a single metric of app engagement, that is, the first PC. For app engagement in the 3-month period, the first PC captures 58% of the variance comprising the 5 engagement indicators, whereas the other 4 components capture at most 16% of the variance. For app engagement in the 6-month period, the first PC captures 65% of the variance, whereas the other 4 components capture at most 14%. According to these numbers, we treat the first PCs as valid metrics of app engagement in the 3- and 6-month periods, respectively. The coefficients for the linear combinations of indicators of app engagement that yield the PCs are (1) *PC_3 months_ = 0.32 accessed pages of learn content + 0.53 meal count + 0.22 weight count + 0.47 activity count + 0.59 completed tasks* and (2) *PC_6 months_ = 0.18 accessed pages of learn content + 0.65 meal count + 0.24 weight count + 0.39 activity count + 0.58 completed tasks* (all counts were log10 transformed before PCA). A median split was then applied to this metric to distinguish between the patients showing lower app engagement and the patients showing higher app engagement. The subsamples of patients with higher and lower app engagement were then matched according to the patient characteristics (ie, gender, age, diagnosis, and start weight) and message exchange between the patient and the coach. Multimedia Appendix 2 shows the statistics for weight outcome, for the generic predictors, and for the indicators of app engagement in the matched subsamples of lower and higher app engagement. The table has an upper part for the 3-month period and a lower part for the 6-month period. This division is necessary because the patients could engage differently with the app in the 2 periods and were categorized differently accordingly. As a consequence, the composition of the matched samples varied between the 2 periods.

**Figure 3:**
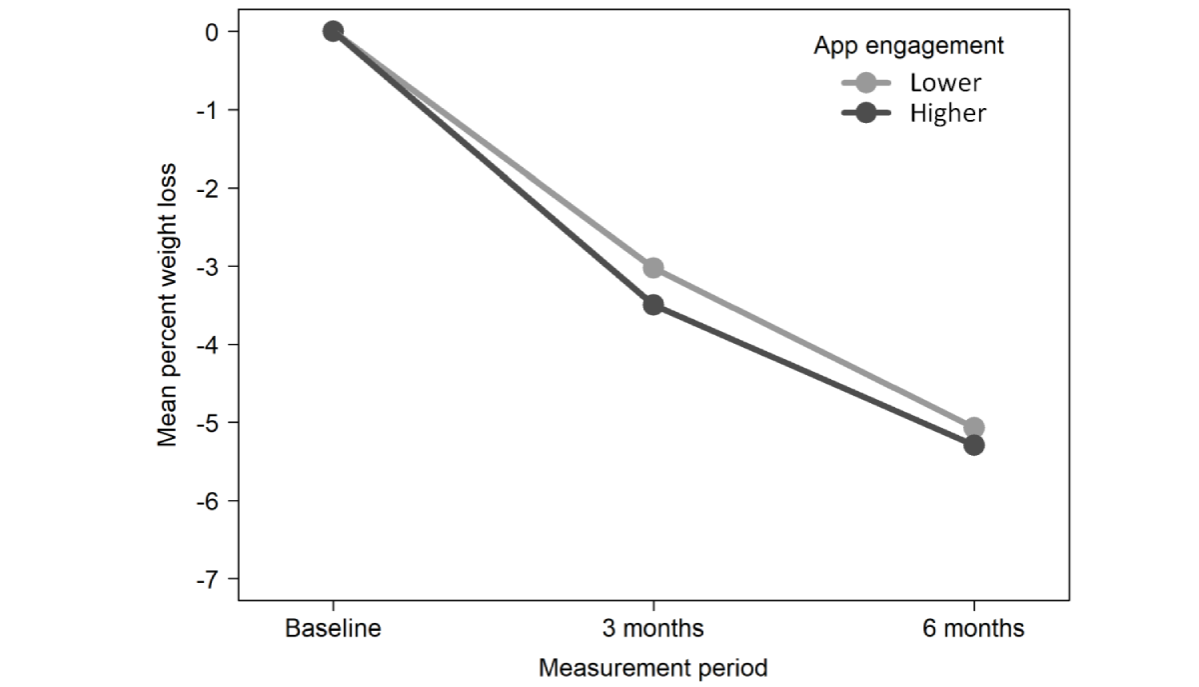
Mean percent weight loss across countries for lower and higher app engagement.

**Figure 4:**
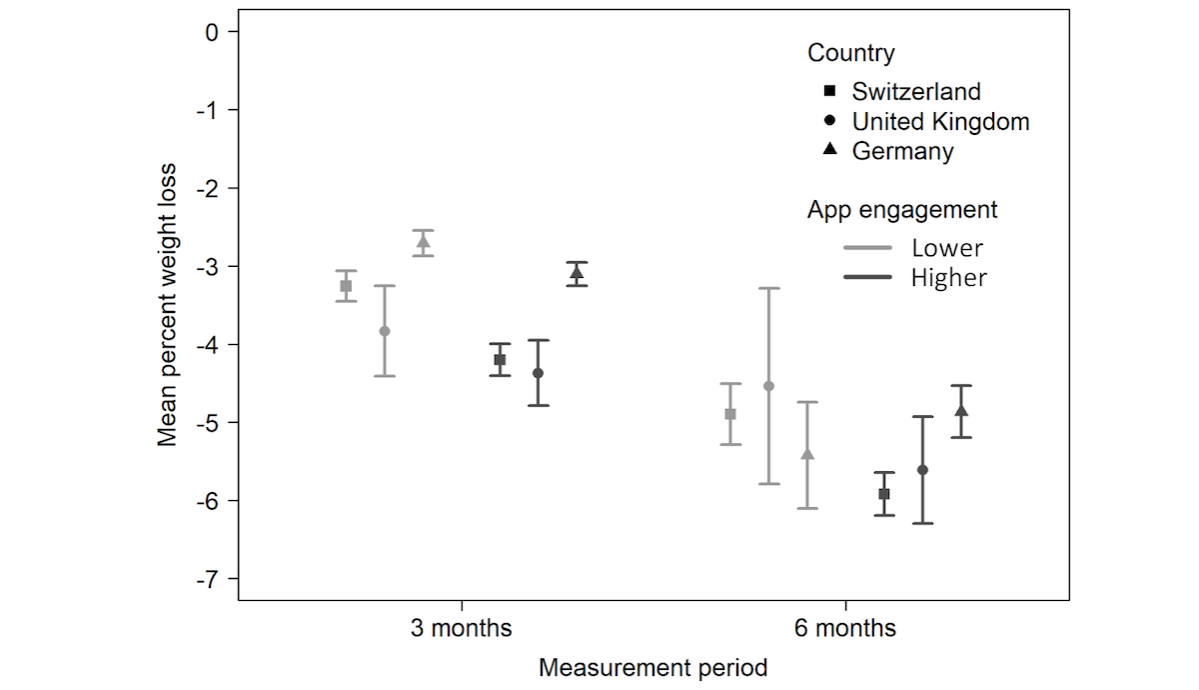
Mean percent weight loss for lower and higher app engagement in 3 countries. Error bars represent 95% CIs.

Descriptively, weight outcome shows stronger average weight loss for higher app engagement than for lower engagement across countries (Figure 3). Furthermore, the same pattern was observed for the data from Switzerland and the United Kingdom, but not for the data from Germany (Figure 4). In fact, stronger weight loss with lower app engagement was observed on average in Germany after 6 months. Furthermore, the generic predictors and the indicators of app engagement show the required pattern. Because of the matching, the averages for the generic predictors vary little between lower and higher engagement, indicating successful matching. Conversely, because of the categorization into lower and higher engagement, the averages of the engagement indicators show higher variation.

### Weight Loss Models

Multimedia Appendix 3 relates to the hypothesized association between percentage weight loss and app engagement after 3 and 6 months of intervention. This association was confirmed in the 3-month period for all countries pooled, for Switzerland, and for Germany. Here, patients with higher app engagement lost significantly more weight than patients with lower engagement (pooled: b_3 months_=–0.34; *P*<.001; Switzerland: b_3 months_=–0.9; *P*<.001; and Germany: b_3 months_=–.34; *P*=.005). In the 6-month period, no significant association between higher app engagement and higher weight loss was found (pooled: b_6 months_=–0.10; *P*=.59), except for 1 coefficient. That is, in the 6-month period, patients with higher app engagement lost significantly more weight only in Switzerland (b_6 months_=–0.9; *P*<.001). For the data from the United Kingdom, the association could not be established (United Kingdom: b_6 months_=–1.06; *P*=.12) and, conflicting with our hypothesis, in individual dietetic therapy in Germany, a positive regression coefficient indicated more weight loss with lower app engagement (Germany: b_6 months_=0.42; *P*=.21). However, a consistently significant predictor across the countries was start weight, indicating that people with higher start weights achieved more weight loss (pooled: b_3 months_=–.03; *P*<.001 and b_6 months_=–.04; *P*<.001; Switzerland: b_3 months_=–0.04; *P*<.001 and b_6 months_=–0.04; *P*<.001; United Kingdom: b_3 months_=–0.04; *P*<.001 and b_6 months_=–0.04; *P*=.006; and Germany: b_3 months_=–0.03; *P*<.001 and b_6 months_=–0.06; *P*<.001). Similarly, in the 3-month period, persons of a higher age achieved more weight loss (pooled: b_3 months_=–0.02; *P*<.001; Switzerland: b_3 months_=–0.02; *P*<.001; United Kingdom: b_3 months_=–0.05; *P*=.002; and Germany: b_3 months_=–0.02; *P*<.001). Interestingly, the number of messages received from the coach yielded no significant effect on weight loss (pooled: b_3 months_=–0.05; *P*=.45 and b_6 months_=0.12; *P*=.37; Switzerland: b_3 months_=–0.07; *P*=.51 and b_6 months_=0.2; *P*=.26; United Kingdom: b_3 months_=0.26; *P*=.37 and b_6 months_=0.8; *P*=.39; and Germany: b_3 months_=–0.02; *P*=.91 and b_6 months_=0.31; *P*=.39), contrasting with significant effects of the number of messages to the coach, at least for the pooled data across countries (b_3 months_=–0.72; *P*<.001 and b_6 months_=–1.12; *P*<.001) and for Switzerland (b_3 months_=–0.57; *P*<.001 and b_6 months_=–1.09; *P*<.001). This may indicate a kind of asymmetrical impact of communication between the patient and the coach on weight loss.

## Discussion

### Principal Findings

App engagement was a consistent predictor of percent weight loss across countries in the 3-month period (except for the United Kingdom) but not in the 6-month period. In the 6-month period, only in the data from Switzerland was higher app engagement associated with higher weight loss. In the data from the United Kingdom and Germany, this association was not statistically significant. In fact, higher app engagement appears to be associated with less weight loss after 6 months in Germany, potentially linked to the focus on individual follow-up consultations, rather than app-based care in that program. Furthermore, in the German real-world setting, the program in some cases extends beyond 6 months, so that not all parts of it may have affected weight outcomes measured at 6 months. Therefore, our hypothesis that app engagement yields weight loss was confirmed in the 3-month period, but not in the 6-months period. Notably, whereas higher numbers of patient messages to the coach were associated with more weight loss in the Swiss data and in the overall data set, this association was not found with the number of messages sent from the coach to the patient. The most consistent predictors for weight loss were start weight and age. Patients with higher start weight achieved significantly stronger weight loss after 3 and 6 months and those of higher age achieved stronger loss after 3 months of intervention.

### Comparison With Previous Work

Our results confirm that early app engagement in blended-care weight management interventions (ie, time period of the first 3 months) is associated with additional weight loss as an outcome. Our contribution shows similarities to a retrospective real-world data analysis of 2113 patients with overweight from 2011 to 2015 [18]. This research revealed the number of weight measurements, activity times, and the number of food logs to be predictors of weight loss after 6 months. Using a multiple of the earlier sample size and separating the analysis for 3 different countries, our study revealed that such behavioral indicators truly predict weight loss, however, only for the 3-month period using aggregated indicators of app engagement.

More similar results originate from a large sample of 251,718 patients from China recorded from 2016 and 2017 [10]. This study showed that the frequency of app usage predicts weight loss after 4 months. A comparable effect of frequency of weighing on weight loss was observed by the same research group in patients who are severely obese with BMI≥35 kg/m² [28]. The obtained weight loss outcomes (overall: –3.24%,–3.26 kg at 3 months and –5.22%,–5.25 kg at 6 months) exceeded the weight loss identified in recent systematic reviews on the efficacy of smartphone-based weight loss interventions. For example, absolute weight loss of –1.99 kg [29] and –2.18 kg [30] for the 3-month period were documented. Moreover, the observed overall weight loss at 6 months is clinically meaningful and in line with the weight loss goals for this time period stipulated in the pertinent national and international guidelines [31,32].

To further improve weight loss as well as the intervention design, it is key to analyze the factors that drive engagement, as per the cohort and at the individual level [8]. Moreover, as earlier research indicated the time dependency of digital intervention elements that contribute to app engagement, the dimension of time needs to be considered in addition to quantitative and qualitative factors of engagement [20]. An in-depth understanding of these factors and how patients respond to them is a precondition for tailored engagement, and thus, personalized interventions.

### Strengths and Limitations

In our study, app engagement was a particularly strong metric, based on recorded app data, which was not prone to data shortage or data loss. Therefore, we consider these data to yield a valid distinction between lower and higher app engagement. In fact, we recommend future use of this metric because it covers 3 behavioral areas known to be crucial in required lifestyle changes in patients with obesity—self-monitoring, self-management, and education. Furthermore, the propensity score matching used to equalize the groups of patients with higher and lower app engagement allowed for successful isolation of the effect of engagement on weight loss. This supports the idea that the observed differences in average weight loss after 3 or 6 months of intervention can be traced back to the differences in app engagement between the 2 groups of higher and lower app engagement in the respective time period. However, there may still be unobserved factors that may account for both quantities, app engagement and weight loss. Only a randomized controlled trial could rule out this possibility. This trial would have to manipulate (not only measure) app engagement and control for confounding variables using randomization [33].

Data collection for this study covered a long period from January 3, 2019, to August 25, 2022. Although we may be confident that this duration leveled out any potential seasonal effects on the study outcome, it was, nevertheless, possible that the 3 programs in Switzerland, Germany, and the United Kingdom underwent structural changes and staff rotation. Moreover, because the 3 programs differed in more than one respect, causal agents within the programs that contribute to app engagement and eventually yield weight loss are difficult to identify. However, the used programs can be considered as standard blended care in the 3 countries, which emphasizes the transferability of results across persons, settings, and time.

### Conclusions

App engagement predicts weight loss in blended-care interventions, with higher engagers losing more weight than lower engagers. Our data consolidate the association between early app engagement and successful weight loss. The results stress that patients may be most receptive in the early phase of a weight loss intervention, opening opportunities for innovative treatment concepts such as just-in-time adaptive interventions supported by chatbot-based digital coaching [34]. Fitting in with this is our secondary result that messages sent by the patient have a stronger effect than messages sent by the coach. Possibly, human-written coach messages could be gradually replaced by automatic messages to maintain the messaging activity of the patient. However, future research should focus on the patient-intervention-fit for these options to be practicable. Furthermore, future research should focus on the reasons for lower app engagement. The potential fear of data exploitation as a known impediment to trust in digital technology [35] may be a tenable explanation. Future research should further scrutinize the differential impact of the components of app engagement [14].

In general, blended-care weight management interventions have surged in popularity in recent years for various reasons. App use for the treatment entails low barriers for most patients, increasing uptake. Digital care furthermore offers clinicians and health coaches real-time insights into the trajectories of important clinical outcomes. Finally, digital care produces comprehensive data sets needed for pivotal insights in health care research. While following the currents of digitization in health care to facilitate clinical delivery and data acquisition in routine care is beneficial, the nascent digital treatment revolution lies in the introduction of digital devices with a manifest effect on health outcomes [36].

## Appendix

Multimedia Appendix 1 STROBE (Strengthening the Reporting of Observational Studies in Epidemiology) checklist cross-sectional studies.

Multimedia Appendix 2 Characteristics of patients with lower and higher app engagement.

Multimedia Appendix 3 Weight loss prediction models using patient characteristics and app engagement as predictors.

## Abbreviations

*ICD-10*: *International Classification of Diseases, Tenth Revision*
MDR: Medical Device Regulation
PC: principal component
PCA: principal component analysis
STROBE: Strengthening the Reporting of Observational Studies in Epidemiology

## Data Availability

This study used real-world data from actual therapeutic care. The data sets generated and analyzed during this study are not publicly available because patient consent only allows for the usage of anonymized data by Oviva for the purpose of this study but precludes making the data publicly available.
