# Supplementary material for: App Engagement as a Predictor of Weight Loss in Blended-Care Interventions: Retrospective Observational Study Using Large-Scale Real-World Data
Source: J Med Internet Res. 2024 Jun 7;26:e45469. doi: 10.2196/45469 (PMC11193074; doi:10.2196/45469)
Supplement: Multimedia Appendix 2 [file jmir_v26i1e45469_app2.pdf]

Predictors 3 months relating to lower and higher app engagement

	All countries n = 15267				Switzerland n = 4570				United Kingdom n = 926				Germany n = 5396			
	lower		higher		lower		higher		lower		higher		lower		higher	
	M	SD	M	SD	M	SD	M	SD	M	SD	M	SD	M	SD	M	SD
**Outcome**																
Percent weight loss 3 months	-3.02	4.84	-3.5	4.29	-3.26	4.79	-4.2	5.01	-3.83	6.31	-4.37	4.56	-2.71	4.36	-3.1	3.95
**Generic predictors**																
Gender[a]	0.71	0.45	0.82	0.38	0.74	0.44	0.74	0.44	0.79	0.41	0.81	0.39	0.8	0.4	0.82	0.39
Age	47.63	13.42	50.79	12.44	49.94	13.47	49.81	13.1	47.98	12.07	48.43	12.19	47.23	12.89	47.52	12.19
E66 Diagnosis[b]	0.68	0.47	0.75	0.43	0.76	0.42	0.76	0.43	0.27	0.44	0.25	0.44	0.74	0.44	0.75	0.43
Start weight	100.24	24.89	101.25	22.92	95.08	19.41	95.19	19.65	125.48	29.66	124.56	24.5	103.69	25.15	103.53	23.76
Log10 Messages from coach 3 months	0.87	0.61	1	0.6	1.07	0.66	1.13	0.76	0.43	0.62	0.42	0.62	0.9	0.45	0.91	0.49
Log10 Messages to coach 3 months	0.12	0.33	0.16	0.41	0.21	0.43	0.28	0.51	0.49	0.59	0.69	0.68	0.03	0.17	0.04	0.17
**Indicators of app engagement**																
Log10 Pages of learn content 3 months	0.28	0.45	0.91	0.7	0.2	0.34	0.52	0.54	0.52	0.57	1.15	0.63	0.39	0.54	1	0.71
Log10 Meal count 3 months	1.31	0.78	2.19	0.26	1.38	0.78	2.19	0.23	1.36	0.79	2.15	0.24	1.36	0.7	2.18	0.26
Log10 Number of weight logs 3 months	0.74	0.33	1.15	0.36	0.75	0.33	1.13	0.36	0.8	0.27	1.07	0.26	0.77	0.34	1.15	0.38
Log10 Activity count 3 months	0.22	0.44	1.15	0.73	0.21	0.43	1.07	0.7	0.27	0.45	0.98	0.76	0.25	0.44	1.17	0.74
Log10 Number of completed tasks 3 months	0.76	0.63	1.86	0.37	0.78	0.65	1.77	0.33	0.6	0.62	1.73	0.39	0.88	0.59	1.89	0.35

[a] Proportion of female patients, [b] Proportion of patients diagnosed with ICD-10 E66

Predictors 6 months relating to lower and higher app engagement

	All countries n = 11839				Switzerland n = 4668				United Kingdom n = 662				Germany n = 4290			
	lower		higher		lower		higher		lower		higher		lower		higher	
	M	SD	M	SD	M	SD	M	SD	M	SD	M	SD	M	SD	M	SD
**Outcome**																
Percent weight loss 6 months	-5.07	6.55	-5.3	5.77	-4.9	5.64	-5.92	5.76	-4.54	6.21	-5.61	5.58	-5.42	7.93	-4.86	5.76
**Generic predictors**																
Gender[c]	0.73	0.45	0.79	0.41	0.7	0.46	0.71	0.45	0.8	0.4	0.79	0.4	0.82	0.39	0.82	0.39
Age	48.03	13.4	50.42	12.74	48.82	13.83	49.54	13.24	49.34	11.83	49.44	12.28	48.29	12.84	49.39	12.66
E66 Diagnosis[d]	0.68	0.47	0.75	0.43	0.73	0.45	0.76	0.43	0.31	0.46	0.31	0.46	0.76	0.43	0.76	0.42
Start weight	99.85	24.85	101.14	23	94.28	19.64	96.22	19.84	122.11	28.02	121.3	23.62	103.28	24.69	102.89	24.49
Log10 Messages from coach 6 months	0.46	0.47	0.78	0.59	0.65	0.49	0.94	0.69	0.18	0.34	0.17	0.32	0.5	0.43	0.54	0.43
Log10 Messages to coach 6 months	0.05	0.19	0.12	0.32	0.07	0.24	0.19	0.4	0.18	0.34	0.3	0.4	0.02	0.13	0.02	0.12
**Indicators of app engagement**																
Log10 Pages of learn content 6 months	0.17	0.37	0.53	0.62	0.12	0.25	0.3	0.41	0.25	0.44	0.65	0.63	0.26	0.48	0.61	0.67
Log10 Meal count 6 months	0.6	0.68	2.12	0.39	0.65	0.7	2.13	0.36	0.69	0.72	2.09	0.37	0.65	0.65	2.07	0.39
Log10 Number of weight logs 6 months	0.3	0.37	0.8	0.5	0.34	0.37	0.83	0.45	0.31	0.35	0.83	0.41	0.28	0.37	0.74	0.53
Log10 Activity count 6 months	0.14	0.38	0.92	0.82	0.13	0.36	0.8	0.79	0.09	0.26	0.84	0.81	0.16	0.4	0.98	0.83
Log10 Number of completed tasks 6 months	0.36	0.47	1.73	0.51	0.33	0.46	1.62	0.51	0.29	0.41	1.65	0.5	0.49	0.51	1.78	0.47

[c] Proportion of female patients, [d] Proportion of patients diagnosed with ICD-10 E66
