# Supplementary material for: App Engagement as a Predictor of Weight Loss in Blended-Care Interventions: Retrospective Observational Study Using Large-Scale Real-World Data
Source: J Med Internet Res. 2024 Jun 7;26:e45469. doi: 10.2196/45469 (PMC11193074; doi:10.2196/45469)
Supplement: Multimedia Appendix 3 [file jmir_v26i1e45469_app3.pdf]

	All countries<br>Percent weight loss after 3 months			All countries<br>Percent weight loss after 6 months			Switzerland<br>Percent weight loss after 3 months			Switzerland<br>Percent weight loss after 6 months			UK<br>Percent weight loss after 3 months			UK<br>Percent weight loss after 6 months			Germany<br>Percent weight loss after 3 months			Germany<br>Percent weight loss after 6 months		
Predictors	*Estimates*	*CI*	*p*	*Estimates*	*CI*	*p*	*Estimates*	*CI*	*p*	*Estimates*	*CI*	*p*	*Estimates*	*CI*	*p*	*Estimates*	*CI*	*p*	*Estimates*	*CI*	*p*	*Estimates*	*CI*	*p*
Gender Female	0.08	-0.10 – 0.26	0.361	-0.21	-0.60 – 0.17	0.280	-0.39	-0.75 – -0.04	**0.031**	-0.47	-1.01 – 0.07	0.085	0.43	-0.48 – 1.35	0.356	0.54	-1.01 – 2.09	0.491	-0.04	-0.36 – 0.28	0.811	-0.60	-1.45 – 0.24	0.160
Age	-0.02	-0.03 – -0.01	**<0.001**	-0.02	-0.03 – -0.00	**0.009**	-0.02	-0.03 – -0.01	**0.001**	-0.01	-0.03 – 0.00	0.087	-0.05	-0.08 – -0.02	**0.002**	-0.02	-0.07 – 0.03	0.460	-0.02	-0.03 – -0.01	**<0.001**	-0.03	-0.05 – -0.00	**0.025**
E66 Diagnosis	-0.11	-0.28 – 0.05	0.175	-0.61	-0.98 – -0.24	**0.001**	0.10	-0.27 – 0.46	0.610	-1.10	-1.68 – -0.52	**<0.001**	-0.05	-0.84 – 0.75	0.909	-0.59	-1.91 – 0.74	0.384	0.04	-0.26 – 0.35	0.783	0.65	-0.14 – 1.43	0.107
Start weight	-0.03	-0.03 – -0.03	**<0.001**	-0.04	-0.05 – -0.03	**<0.001**	-0.04	-0.05 – -0.04	**<0.001**	-0.04	-0.05 – -0.03	**<0.001**	-0.04	-0.05 – -0.03	**<0.001**	-0.04	-0.06 – -0.01	**0.006**	-0.03	-0.03 – -0.02	**<0.001**	-0.06	-0.07 – -0.04	**<0.001**
Log10 Messages from coach until 3 months	-0.05	-0.16 – 0.07	0.451				-0.07	-0.26 – 0.13	0.509				0.26	-0.31 – 0.84	0.370				-0.02	-0.27 – 0.24	0.906			
Log10 Messages to coach until 3 months	-0.72	-0.92 – -0.53	**<0.001**				-0.57	-0.87 – -0.27	**<0.001**				-0.09	-0.66 – 0.47	0.749				-0.82	-1.45 – -0.18	**0.012**			
Log10 Messages from coach until 6 months				0.12	-0.15 – 0.40	0.374				0.20	-0.15 – 0.54	0.260				0.80	-1.04 – 2.64	0.394				0.31	-0.40 – 1.02	0.393
Log10 Messages to coach until 6 months				-1.12	-1.59 – -0.65	**<0.001**				-1.09	-1.66 – -0.53	**<0.001**				0.73	-0.77 – 2.22	0.340				-0.88	-2.80 – 1.04	0.367
Higher app engagement until 3 months	-0.34	-0.49 – -0.19	**<0.001**				-0.90	-1.18 – -0.62	**<0.001**				-0.55	-1.26 – 0.15	0.124				-0.34	-0.58 – -0.11	**0.005**			
Higher app engagement until 6 months				-0.10	-0.45 – 0.26	0.592				-0.90	-1.38 – -0.42	**<0.001**				-1.06	-2.41 – 0.28	0.121				0.42	-0.24 – 1.08	0.213
(Intercept)	1.06	0.54 – 1.57	**<0.001**	0.24	-0.90 – 1.37	0.685	2.25	1.14 – 3.37	**<0.001**	0.84	-0.84 – 2.52	0.328	3.29	0.49 – 6.10	**0.021**	0.20	-4.82 – 5.21	0.939	1.11	0.23 – 2.00	**0.014**	2.05	-0.31 – 4.42	0.088
Observations	15264			5529			4570			2505			924			356			4289			1685		
R^2 / R^2 adjusted	0.035 / 0.034			0.032 / 0.031			0.041 / 0.040			0.049 / 0.046			0.057 / 0.050			0.045 / 0.025			0.032 / 0.030			0.046 / 0.042		
